# The State of the Art in Biodefense Related Bacterial Pathogen Detection Using Bacteriophages: How It Started and How It’s Going

**DOI:** 10.3390/v12121393

**Published:** 2020-12-04

**Authors:** Shanmuga Sozhamannan, Edward R. Hofmann

**Affiliations:** 1 National Security Science & Technology, Management Advisory Services, Logistics Management Institute, 7940 Jones Branch Drive, Tysons, VA 22102, USA; shanmuga.sozhamannan.ctr@mail.mil; 2 Defense Biological Product Assurance Office (DBPAO), Joint Program Executive Office (JPEO) for Chemical, Biological, Radiological and Nuclear Defense (CBRND) Joint Project Lead (JPL) CBRND Enabling Biotechnologies (EB), 110 Thomas Johnson Drive, Suite 250, Frederick, MD 21702, USA; 3 EXCET, Inc., 6225 Brandon Ave #360, Springfield, VA 22150, USA; 4 US Army Combat Capabilities Development Command, Chemical Biological Center, 8908 Guard St, E3831, Edgewood, MD 21010, USA

**Keywords:** bacteriophage, phage detection, diagnostics, biothreat, bioterrorism

## Abstract

Accurate pathogen detection and diagnosis is paramount in clinical success of treating patients. There are two general paradigms in pathogen detection: molecular and immuno-based, and phage-based detection is a third emerging paradigm due to its sensitivity and selectivity. Molecular detection methods look for genetic material specific for a given pathogen in a sample usually by polymerase chain reaction (PCR). Immuno-methods look at the pathogen components (antigens) by antibodies raised against that pathogen specific antigens. There are different variations and products based on these two paradigms with advantages and disadvantages. The third paradigm at least for bacterial pathogen detection entails bacteriophages specific for a given bacterium. Sensitivity and specificity are the two key parameters in any pathogen detection system. By their very nature, bacteriophages afford the best sensitivity for bacterial detection. Bacteria and bacteriophages form the predator-prey pair in the evolutionary arms race and has coevolved over time to acquire the exquisite specificity of the pair, in some instances at the strain level. This specificity has been exploited for diagnostic purposes of various pathogens of concern in clinical and other settings. Many recent reviews focus on phage-based detection and sensor technologies. In this review, we focus on a very special group of pathogens that are of concern in biodefense because of their potential misuse in bioterrorism and their extremely virulent nature and as such fall under the Centers for Disease and Prevention (CDC) Category A pathogen list. We describe the currently available phage methods that are based on the usual modalities of detection from culture, to molecular and immuno- and fluorescent methods. We further highlight the gaps and the needs for more modern technologies and sensors drawing from technologies existing for detection and surveillance of other pathogens of clinical relevance.

## 1. Introduction

Bacteriophages, also known as phages, are ubiquitous viruses that infect bacteria and archaea. Like their eukaryote relatives, phages can consist of either double-stranded or single-stranded DNA or RNA genomes. This diversity of phages can be seen in both their morphology and genome architecture, with genome size that ranges from ~3–500 kb. First described independently in 1915 by Frederick Twort and in 1917 by Felix d’Herelle, we now know phages are the most abundant taxon in the biosphere with an estimated 10^31^ viral particles. Since their discovery, phages have had an enormous impact on our understanding of genetics, molecular biology, bacterial evolution, and marine and soil ecology. Furthermore, the study of bacterial defenses against phages have led to powerful scientific tools including restriction enzymes and the revolutionary gene editing tools of the CRISPR-Cas system. The discovery of restriction enzyme system was awarded the Nobel Prize for Physiology/Medicine in 1978 and in 2020, CRISPR-Cas-based gene editing application was awarded the Nobel Prize in Chemistry.

Phages were the first to have their entire genomes sequenced and, indeed, the use of phage derived products such as T7 DNA polymerase, T4 DNA ligase, M13 vectors were critical in whole genome sequencing projects from phages to humans. Phages themselves have played an immense role in developing tools and methods for pathogen detection and identification. From the Center for Disease Control and Prevention’s (CDC) gold standard for “phage typing” bacterial isolates to the development of the phage display system for discovery of binding peptides and antibodies, researchers have found creative ways to exploit phages for novel pathogen detection platforms. There are many reviews on the use of phages for pathogen detection using different assays, platforms and biosensors [1,2,3,4,5,6,7,8,9]. This review will focus on how phages have been exploited for detection of those pathogens that are of most concern due to their history and potential for use as biowarfare and bioterrorism agents.

**Figure 1:**
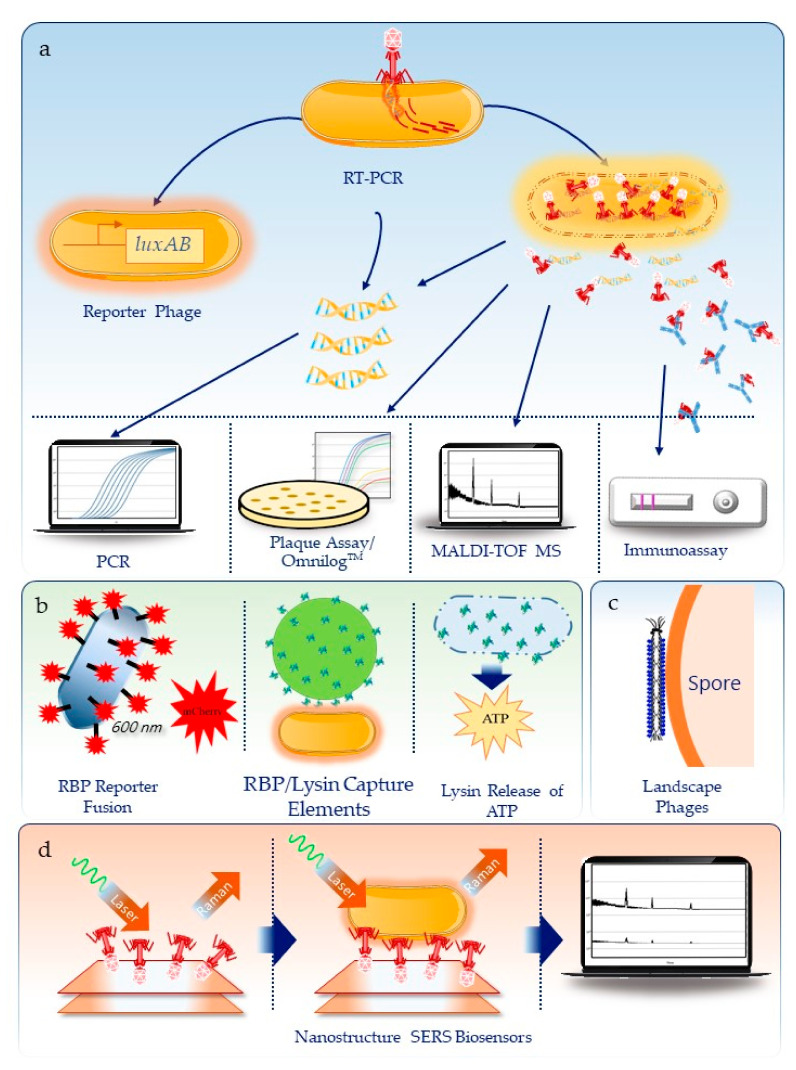
Summary of approaches used to detect biothreats using bacteriophage. (**a**) Methods dependent on the infection and metabolic activity or lysis of the bacteria cell, including reporter phages, molecular and immuno-detection for phage RNA/DNA and proteins, respectively, detection of phage proteins by mass spectrometry, and the traditional plaque assay or real-time, high throughput detection on the Omnilog™. (**b**) Use of phage components, including receptor binding proteins (RBP) reporter fusions to reporter molecules like florescent proteins, use of RBPs or lysins as capture agents on Qdots or gold nanoparticles, or use of lysins to release ATP that can be measured using a luminescent enzyme assay. (**c**) Engineered filamentous phages for spore detection and (**d**) as capture agents on plasmonic nanostructures coupled with surface enhanced Raman spectroscopy (SERS) for Brucella detection.

Historically, the use of phages in bacterial detection started with phage typing schemes for strains of specific pathogens such as *Escherichia coli*, *Klebsiella*, *Shigella*, *Salmonella*, and *Vibrio cholera* [10]. Over time, more diverse and innovative approaches have been taken. The state of the art in phage-based detection of biodefense pathogens is illustrated in Figure 1 and summarized in Table 1 along with key features such as limit of detection and time to result. In addition, the whole genome sequence features of phages that infect different biodefense pathogens are provided in a Supplementary table. In the following sections, we elaborate on the detection modalities currently available for individual pathogens.

## 2. Use of Pathogen Specific Phages

### 2.1. Anthrax

Following the 2001 anthrax letter attacks in the United States, there has been a renewed emphasis on early warning and rapid detection of the deadliest pathogens on the CDC Select Agent list (https://www.selectagents.gov/SelectAgentsandToxinsList.html). *Bacillus anthracis* is the bacterium that causes the infectious disease anthrax and is a Gram positive spore forming pathogen. Anthrax spores can be produced at high concentrations and disseminated efficiently and have been weaponized for use in bioterrorism. For these reasons it is an ideal biological weapon of mass destruction. Left untreated for more than 24 h, anthrax is usually fatal. Diagnosis is difficult symptomatically and it was traditionally identified by clinical, microbiological, and morphological methods.

**Culture-based methods:** Use of phages for detection of *B. anthracis* vegetative cells dates back to the 1950s with the identification of a phage variant γ, which could infect and lyse encapsulated smooth forms from active infections of *B. anthracis* and differentiate between *B. anthracis* and *B. cereus* strains [11]. This assay was further refined and eventually FDA approved in 2005 for clinical diagnosis and is used by the Laboratory Response Network (LRN) [35,36]. Specificity of the assay is very high with very few non-*B anthracis* strains being susceptible to infection. With over a hundred *B. anthracis* strains tested, very few have been found to be resistant to phage γ. One of the disadvantages of culture-based γ phage assay is that it takes 20 h for the confirmation from visualizing clear plaques. A faster high throughput Omnilog^TM^-based indirect phage assay has also been reported for the real time detection of *B. anthracis* [12].

**Phage specific PCR and immunoassay methods:** In an attempt to speed up and reduce the time ‘from sample to result’ of the assay, a real-time PCR assay was developed to detect the amplification of phage DNA [13]. This reduced the detection time to 5 h and had the added benefit of determining the viability of the bacteria in test sample since only metabolically active *B anthracis* cells would allow for phage DNA amplification. Alternatively, a method that detects the initial burst of γ phage RNA upon infection by RT-PCR allows for shortened incubation times and the ability to determine antibiotic sensitivity due to the suppressed metabolic state in the presence of antibiotics [14]. Another approach to using phage amplification as a means to detect vegetative *B. anthracis* cells is to use phage specific antibodies in an immunoassay format. Using the lateral flow immunoassay (LFI) format, Cox et. al. was able to detect 8 × 10^5^ cells in as little as 2 h and down to 1.5 × 10^5^ cells in 4 h [15].

**Phage reporter assays:** An indirect measurement of phage growth to detect viable *B. anthracis* cells is the use of reporter phages. One of the first attempts on this idea was to engineer the parental phage of γ, phage Wβ [16]. Two phage genes considered non-essential were replaced with the bacterial luciferase genes *luxAB* from *Vibrio harveyi*. As expected, the reporter phage is inert and unable to express the *luxAB* genes until the phage is able to attach and inject its genome into the host cell. At this point, the bacterial host transcriptional and translational machinery is taken over by the phage and upon expression of phage genes will also express the luciferase enzyme which produces bioluminescence upon the addition of a substrate. With this reporter phage, vegetative *B. anthracis* cells could be detected down to about a thousand cells/mL, 60 min post infection and as fast as 16 min at higher cell densities. By using the temperate phage Wβ, the luciferase enzyme can accumulate within the bacterial host to increase the amount of bioluminescence produced, thus increasing the sensitivity of the assay. The drawback of using the phages γ and Wβ is that they can infect only vegetative cells and not spores; in order to detect encapsulated *B. anthracis* spores they need first to be cultured in germinating conditions to allow for infection, thus adding additional step and time to the assay. The Wβ reporter phage displayed 100% inclusivity (38 of 38 strains) for *B. anthracis* [37]. Six out of the 126 non-*anthracis Bacillus* near neighbors such as *B. cereus*, *B. mycoides*, and *B. weihenstephanensis*, (95% specificity) elicited bioluminescent signals, albeit at reduced intensities compared to *B. anthracis*. Species outside the *Bacillus* genus tested negative for bioluminescence. After protocol optimization, this phage was able to detect germinated *B. anthracis* spores in a range of spiked matrices including blood, ground beef, milk, and other dairy products [37,38]. After optimization of the reporter, increased sensitivity allowed for detection of spores in environmental soil samples and water samples from pond, lake or brackish waters, demonstrating a wide range of application for spore detection [39,40].

**Receptor binding proteins (RBPs) as tools for detection:** The first step in phage life cycle is the attachment of the phage to its cognate bacterial host through interaction between a phage component known as the receptor binding protein or RBP and the bacterial receptor on the cell surface. RBPs provide the same specificity as the whole phage in targeting the host and RBPs have been successfully used for detection of *B. anthracis.* In tailed phages, it is the tail fiber protein that is most often responsible for determining the specificity of the phage. RBPs can be used as a detection reagent independent of the whole phage. One approach is to fuse RBPs to fluorescent reporters. For phage γ, fusing the RBP (gp14) to green fluorescent protein (GFP) allows for fluorescent labeling of *B. anthracis* [17]. Using a bioinformatics approach, Braun, P. et. al. were able to predict a putative RBP (P28 + P29) encoded by phage AP50c based on genomic synteny and small identical stretches of protein sequence with the RBP (P24) polypeptide sequences from phage Wip1 [18]. Phages AP50c and Wip1 are similar phages belonging to the *Tectiviridae* family. Using the RBP (gp14) sequence from phage γ, they were also able to identify another putative RBP of the prophage λBa03 present on the *B. anthracis* genome. These RBPs fused to mCherry fluorescent proteins were evaluated for their ability to bind to and detect cultured *B. anthracis* cells by fluorescence microscopy. Full length as well as truncated versions of the RBPs were evaluated and RPB::mCherry reporters from Wip1, AP50c, and truncated λBa03 were found to work best in terms of producing fluorescence signals. Bacterial detection was time and growth phase dependent after germination from spores and only vegetative cells could be detected. Inactivation of *B. anthracis* cells by heat, formaldehyde or peracetic acid did not prevent the detection of several virulent strains using the RBP reporters but sensitivity varied depending on inactivation method. Presence of the poly-γ-D-glutamic acid (PGA) capsule found on cell surface did not prevent detection of virulent strains. Specificity of detection of *B. anthracis* was good; of the 56 non-*anthracis Bacillus* spp. strains tested, most were not bound by any of the three RBP:: mCherry proteins at all, and a small number showed only marginal binding.

**Phage components as tools for detection:** Another approach for detection of *B. anthracis* involved using components of the phages known to specifically target their bacterial host. The first example of this was the use of the γ phage protein PlyG lysin [19]. Gamma phage uses the PlyG to hydrolyze the peptidoglycan components of the cell wall and lyse the cell to release progeny phage. Addition of purified PlyG externally to a *B. anthracis* containing sample, rapidly lysed the bacteria, releasing the cellular ATP. Upon further addition of the luciferase enzyme and the substrate luciferin, as with the reporter phage example described above, light is emitted, allowing for detection of as few as 100 cells [19].

The PlyG protein has also been used for its binding properties and a non-catalytic truncated version was demonstrated to be a capture agent for the detection of *B. anthracis*, both encapsulated and non-encapsulated cells [20]. The C-terminal region of the protein is responsible for specific binding to the cell wall of *B. anthracis* but do not bind to *B. subtilis*, *B. cereus*, *B. thuringiensis*, and three other *Bacillus* species when used in a dot blot format [20]. In another study, short 10–20 amino acid peptides from the C-terminal end of PlyG were shown to bind to *B. anthracis* using the same dot blot assay as used with the truncated protein [21]. By coupling one of the peptides with Quantum dots (Qdots), *B. anthracis* cells could be detected in spiked plasma by fluorescence microscopy or by plate reader with the same specificity as the phage γ assay [22].

**Engineered phages as tools for detection:** Additional peptides that can be used in the Qdot assay for *B. anthracis* detection were discovered by employing a commercial M13 phage display peptide library against *B. cereus* 4342, which is a rare strain also susceptible to phage γ [41]. These peptides BBP-1 and BBP-2 were specific to *B. anthracis* and *B. cereus* 4342 and did not bind to other *B. cereus* or *B. thuringiensis* strains. Based on 2D gel electrophoresis, immunoblotting and peptide extraction and sequencing the authors hypothesize that the BBP-1 and BBP-2 peptides bind to the S-layer protein EA1 from these specific *Bacillus* strains. EA1 gene diversity is quite extensive and it is possible that the EA1 in strains such as *B. cereus* 11778 is diverged enough to preclude binding by these peptides. Comparison of the EA1 gene sequences of *B. cereus* 11778 to *B. anthracis*, *B. cereus 4342*, and other strains can potentially reveal motifs critical for binding of the peptide.

One of the common criticisms about phage-based detection is that it is useful only for bacterial detection and cannot be used for detection of other agents such as spores, viruses, and toxins. However, it is possible to engineer specificities for these non-bacterial agents in a phage-based platform. For example, bioengineering of phages is a powerful tool as demonstrated by the use of the filamentous phage display system of M13 phage for antibody discovery. Many recent reviews describe the application of the M13 phage display system [42,43,44,45,46,47]. One variation of the phage display approach is the landscape phages which are also filamentous phages but instead of fusing scFv or peptide libraries to the pIII minor coat protein, the peptide libraries are fused to the pVIII major coat protein. In phage fd, there are thousands of copies of pVIII repeated along the phage surface [48,49,50]. This allows creating libraries that express the peptides on thousands of copies of major coat protein in a fixed conformation and can be screened for specific binding peptides to a targeted structure. These phages have been used as substitutes for antibodies [48,51]. This approach was used to identify peptide sequences that could bind to *B. anthracis* spores in an ELISA format. Three of these peptides were evaluated for their specificity using a co-precipitation assay and while distant *Bacillus* spores weakly bound to these phages, there was stronger cross-reactivity to closer *Bacillus* relatives of *B. cereus* and *B. thuringiensis* [52]. One of these phages was used as the capture reagent for magnetoelastic resonating particles. Detection of *B. anthracis* spores was concentration dependent down to 10^3^ spores/mL. Scanning electron micrographs showed spores bound to the phage coated particles but not the uncoated particles. When other *Bacillus* spores *B. cereus* and *B. megaterium* were mixed in, there was some cross-reactivity consistent with the earlier co-precipitation studies [23]. Similar approaches can be taken to develop phages that are specific for viruses and toxin detection.

### 2.2. Plague

*Yersinia pestis* is the etiological agent of the plague. It is a zoonotic disease that affects rodents and can be transmitted from animal to animal by fleas, which happens to be the most common mode of transmission to humans [53]. When bitten by a *Y. pestis* infected flea, humans develop swollen lymph nodes, or buboes, that can develop into septicemia or occasionally into pneumonia. Pneumonic plague infections are transmissible between humans through respiratory droplets and is highly virulent. The three great plagues have been well described elsewhere but today plague is still endemic in certain parts of the globe including the Democratic Republic of the Congo, Madagascar, and Peru. *Y. pestis* is also considered a Category A pathogen with great potential for use in a bioterrorist attack due to its rapid disease progression and high mortality rate. *Y. pestis* grows slowly and diagnosis by culturing methods can take 2–5 days. Alternatively, a positive diagnosis can be determined by observing a 4-fold difference in antibody titers to F1 capsular antigen [54]. Rapid test using ELISA, LFI, PCR, or real-time PCR have been developed to speed up the detection or diagnosis of plague [55,56,57,58,59,60,61,62].

**Culture-based detection:** Similar to γ phage, the CDC recommended *Y. pestis* specific phage for identification and plague diagnosis is φA1122 [24]. As with γ phage, φA1122 is used in the plaque assay format by the CDC, World Health Organization and U.S. Army Research Institute of Infectious Diseases [55]. φA1122 is able to infect most *Y. pestis* strains tested and will only infect the near neighbor *Y. pseudotuberculosis* under certain temperature constraints [24,55,63]. Two other phages are used for plague diagnosis, the Pokrovskaya phage and the L-413C phage [25,26].

**Phage molecular assay:** Sergueev et. al. developed a quantitative real-time PCR (real time-qPCR) assay for φA1122 and L-413C that could be used to detect replicating phage DNA in live *Y. pestis* with a sensitivity of 10^3^ cfu/mL to 10^5^ cfu/mL or one bacterium to 100 cfu per qPCR sample, respectively [25]. This assay proved to be suitable for both lab-based (LightCycler 2.0) and field-deployable (JBAIDS) real time-qPCR systems [26]. The assay was sensitive in simulated blood specimens, serum, and organ samples. Bacteriophage φA1122 showed some degree of amplification on 4 of the 20 *Y. pseudotuberculosis* strains tested at 28 °C. The specificity of this assay was increased to almost 100% by incubating the bacteria at 24 °C. φA1122 did not lyse any of the 17 *Y. enterocolitica* strains tested. L-413C demonstrated greater specificity, regardless of temperature, but was less sensitive for *Y. pestis* detection.

**Reporter phage assay:** The φA1122 phage has also been used to develop a reporter phage by inserting the *luxAB* genes into a non-coding region downstream of the A1, A2, A3 promoters, which are highly active early during infection [27]. In log phase cultures grown at either 28 °C or 37 °C the production of bioluminescence signal is rapid (<15 min) and has a sensitivity of ~800 cells on a plate reader. To demonstrate its clinical relevance, serum samples spiked with *Y. pestis* were evaluated for production of signal upon addition of the reporter phage. Serum did increase the time to detect and damped the strength of signal but detection in less than an hour was possible. Furthermore, blood samples spiked with *Y. pestis* and diluted with LB or transferred to commercial blood culture bottles were compatible for detection with the reporter phage despite a reduction in overall bioluminescence. Cultured samples were detected in as little as 20 min by the reporter phage. Antibiotic susceptibility can also be determined with the application of the reporter phage as the strength of signal diminishes in the presence of antibiotics in a dose dependent manner [28]. After optimizing the reporter phage by integrating the *luxAB* genes under control of the major capsid gene promoter gp10, this new reporter phage allowed for a more rapid and greater dynamic range of detection. After adding phage 1-2 h after adding either chloramphenicol, tetracycline, or streptomycin, bioluminescence was measured. As bioluminescence production is dependent on cell growth, there was an inverse correlation of signal to antibiotic susceptibility [28].

**RBP protein fusions as tools for detection:** As described before with *Bacillus* phages, fluorescent protein fusions to the RBP of the *Y. pestis* phages φA1122 (gp17) and L-413C (gpH) have been used to detect the attenuated EV76 strain of *Y. pestis* species by fluorescent microscopy [29]. This detection capability was dependent on the temperature and time of the culture conditions. Both RBP fusions were able to differentiate *Y. pestis* from other related risk group 2 pathogens *Y. pseudotuberculosis*, *Y. enterocolitica* subsp. *enterocolitica* and *Y. enterocolitica* subsp. *palearctica*, *Y. wautersii*, and *Y. similis* when grown at 28 °C. However, when grown at 37 °C, the φA1122 RBP fusion could detect *Y. pseudotuberculosis,* consistent with the ability of φA1122 to form plaques on *Y. pseudotuberculosis* when grown at this temperature. Therefore, the authors suggest that the L-413C RBP fusion would be better suited for clinical diagnosis. Finally, the process of inactivation by heat, peracetic acid, ethanol, and paraformaldehyde (PFA) was evaluated in this study. It was determined that 4% PFA was ideal for inactivation and used to demonstrate detection of six different risk group 3 strains of *Y. pestis* [29].

**Phage protein-based assay:** Another application of using φA1122 as a surrogate agent for rapid *Y. pestis* identification is the use of phage proteins detected by matrix-assisted laser desorption/ionization time-of-flight mass spectrometry (MALDI-TOF MS) [30]. MALDI-TOF MS has been a valuable tool for rapid bacterial detection but often requires pure cultures grown overnight or more under specific conditions. Infection and detection by MS of an increase in phage proteins during the phage life cycle serves as a surrogate for bacterial detection and eliminates the need for overnight culturing thereby reducing time to result. A drawback to this approach is the need for repetitive, time-consuming sample preparations and analysis over the course of phage infection to determine the detection limit of the phage proteins. However, using a modified version of the Payne and Jansen phage therapy model, specific equations and information such as burst size and time can be used to predict when the phage will be detectable by MALDI-TOF MS in the presence of various concentrations of bacteria and multiplicities of infection (MOI) [30]. The starting concentration of phage used was based on the experimental limit of detection in order to differentiate between the inoculating phage and their progeny. Modeling φA1122 replication in *Y. pestis* demonstrated how these models can be used to predict when the phage 15.8 kDa capsid assembly protein can be detected and effectively reducing the time to detection compared to detecting the bacteria directly. However, time to detection is dependent on the MOI (concentration of both phage and bacteria) with the most rapid detection of an hour in the presence of 10^7^ *Y. pestis* cells [30]. It is noteworthy that the limit of detection of purified phage needs to be determined for each phage and host model for a particular MALDI-TOF MS system.

### 2.3. Brucella Species

Brucellosis is a debilitating and incapacitating bacterial zoonotic disease that infects hundreds of thousands of people all across the globe. In addition, it causes reproductive losses in livestock each year [64,65,66]. The four main species of *Brucella* that cause disease in humans and livestock are, *B. melitensis*, *B. abortus*, *B. suis*, and *B. canis*. *Brucella* spp. and were weaponized during the Cold War and today the CDC still classifies *B. melitensis*, *B. abortus*, and *B. suis* as Category B Select Agents. Diagnosis of brucellosis is challenging as symptoms are not specific and the infection can affect many organs and systems. Laboratory diagnosis usually consist of three modalities: culture, serology, and PCR [67,68,69,70,71,72,73,74,75]. All of these tests have their own set of challenges from long culture times, delayed and false positive serology results, and false negative PCR results due to low bacteremia or post antibiotic treatment and symptomatic false positives, as well as PCR inhibitors in blood and tissues [76,77]. Phage-based testing using lytic phages specific for *Brucella* has been around for decades [31,33].

**Culture-based phage assay:** Brucellaphages Tbilisi (Tb), Firenze (Fz), Weybridge (Wb), S708, Berkeley (Bk), R/C, and Izatnagar (Iz) are used at two different concentrations for identification of *Brucella* at both genus and species level, including differentiation between smooth and rough strains of *B. abortus*, *B. suis*, *B. melitensis*, *B. neotomae*, *B. canis*, and *B. ovis* [10,31,32]. As with other phage lysis assays, this is a multi-day assay requiring pure cultures of Brucella isolates from samples

**Phage molecular assay:** Similar to *Y. pestis* phages, Brucella phages can be used for indirect detection of Brucella spp. Although not as quick as the *Y. pestis* phage PCR assays, the qPCR assay using phage S708 could detect live *B. abortus* S19 cells at concentration from 10^6^–10^8^ CFU/mL in 24 h after the addition of phage to the culture. Extending this incubation time to 48 h increased detection sensitivity to 10^3^–10^5^ CFU/mL range and 72 h 1–100 cells/mL range [78]. A detection sensitivity of one cell per mL of test culture is a remarkable sensitivity considering the low concentration of bacteria in early Brucellosis. Four other phages, Bk, Fz, Tb, and Wb, also showed robust sensitivity as a surrogate qPCR marker for live Brucella. Phage Bk has the ability lyse many smooth Brucella spp. and therefore would be suited for diagnosis of human brucellosis. Testing on samples of near neighbor bacterial species *O. anthropi* and *A. felis* as well as the serological relevant cross reactive species *Y. enterocolitica* serovar O:9, demonstrated the specificity of Bk phage for Brucella spp. Mixing in the other species dampened the qPCR assay for Bk but did not inhibit it adversely for good detection. The assay did not detect phage amplification in several Brucella spiked commercial sheep blood products; so, the assay method had to be modified to remove suspected inhibitors of Brucella growth before qPCR amplification of phages could be achieved [78].

**Phage biosensor-based detection:** The detection of Brucella using phages has also been demonstrated using biosensors that employ surface-enhanced Raman scattering (SERS) technology combined with multilayer octupolar nanostructures [79]. By functionalizing these nanostructures with the Brucella phage Tb using 4-mercaptobenzoic acid, the authors were able to specifically detect inactivated or live *B. abortus* at concentrations of 10^4^ CFU/mL and the ability to take measurements at the single cell level in less than an hour. It is unclear how this biosensor would perform with clinical or environmental samples since the samples of *Brucella* were from cultures re-suspended in water. Another SERS substrate made of plasmonic nanocavities in a photonic quasicrystal arrangement allowed for the detection of femtomolar amounts of Tb but the authors did not test the ability of this biosensor to detect bacteria [80].

### 2.4. Burkholderia

The gram-negative bacterium *Burkholderia pseudomallei* is an opportunistic pathogen that causes the life-threatening infection melioidosis. It is endemic to tropical and subtropical regions of the world and recent studies suggest that it is more widespread than previously thought [81,82,83]. Melioidosis is known to have mortality rates ranging from 30–60% and in the U.S. it is considered to be a Category A select agent due to its aerosol transmissibility and infectivity, severity, and world-wide distribution. Current treatment for the disease involves a strict 20 week regimen of antibiotics, but even with this, relapse and mortality rates remain high [34]. More troubling, *B. pseudomallei* is intrinsically resistant to many antibiotic treatments, particularly aminoglycosides, due to an efflux pump mechanism that works in concert with other resistance mechanisms present in this organism to further elevate multidrug resistance [84].

**Protein-based phage assay:** As described for *Y. pestis*, in silico prediction of phage amplification combined with MALDI-TOF MS has been used to detect the presence of live *B. pseudomallei*. Using the lytic phage φX216, three major proteins are detectable corresponding to phage tail protein, major capsid protein and its doubly charged ion [85]. After determining the limit of detection of the phage proteins in purified phage samples, this model accurately predicted the detection of phage proteins in 2 h at bacteria concentrations of 3.2 × 10^5^ CFU/mL using phage inoculum concentrations below the pre-determined limit of detection. As described above, the need for metabolically active cells for phages to replicate can be leveraged to determine antibiotic sensitivity. The authors were able to demonstrate phage proteins could not be detected in a sensitive *B. pseudomallei* strains in the presence of ceftazidime but when an isogenic antibiotic resistant strain was used, phage proteins, but not bacterial proteins, were detectable in the 2 h time frame predicted in silico, despite the presence of antibiotics.

### 2.5. Future Directions

The current Gold Standard for pathogen detection continues to be PCR. The analytical sensitivity of PCR is extremely high thanks to the huge amplification of the target nucleic acid segment; theoretically a single target copy can be amplified to billion copies in 40 cycles. The analytical specificity of PCR is largely dependent on target selection, i.e., present in target pathogen and not present in other organisms. Among the immunoassay category, LFI is the predominant method for pathogen detection. They are faster, cheaper and easy to use. The analytical sensitivity of LFI is relatively low compared to PCR due to the fact that there is no amplification of the target antigen and is determined by the kinetics of antigen-antibody interactions used in the assay. Similar to PCR, the specificity of the assay is largely determined the presence of the antigen only in the target pathogen and absence in other organisms. Both PCR and LFI do not discriminate live or dead bacterial pathogen. The sensitivity of phage-based assay is largely determined by the detection modality of the assay, i.e., methods such as fluorescence microscopy can detect single target pathogen. If the assay is designed to detect the first step in phage’s life cycle which is adsorption of the phage particle to the cell surface, it can be faster than other methods. The kinetics of interaction of phage-bacteria has not been compared to antigen-antibody interactions. However, both involve protein-protein interactions and are expected to be quite comparable. The specificity of phage-based assay is quite exquisite due to coevolution of the pair. Hence, the phages are extremely specific to the target host species and sometimes even to the strain level. There are exceptions to this rule where a phage specific for a particular species can infect strains of near neighbor species most likely due to the presence of the phage receptor gene or its functional variant.

There are many more examples of how phages are used for pathogen detection for non-biothreat organisms that are well reviewed [2,3,4,6,7,8]. This is in part due to the greater interest in bacteria that cause food borne illness and other common bacterial pathogens that are easier to work with in the laboratory and do not have biosecurity concerns. If we are to take advantage of these phage-based approaches for biodefense, there needs to be more investment into the discovery of phages for biothreat agents. For example, there are no known virulent or temperate phages for the causative agents for Q fever, *Coxiella burnetii*. The chromosome does not contain any prophage sequences nor are there any CRISPR arrays in all except potentially one strain, *Coxiella burnetii str. Namibia*, suggesting most *Coxiella* spp. may not have needed to evolve a defense against phages [86,87,88]. There are also no known natural phages for *Francisella tularensis.* However, the *F. tularensis.* subsp. *novicida* strain U112 genome has both a putative phage and CRISPR/Cas system [89]. Other *Ft.* subsp. lack a CRISPR array do not appear to have a functional CRISRP/Cas system according to the CRISPR-Cas++ database.

Since the dawn of next generation sequencing about 15 years ago, there has been an explosion of whole genome sequences of many biodefense pathogen and near neighbors. Systematically analyzing whole genome sequences for the presence of prophages can expand the repertoire of phages that can be potentially used for detection and diagnostics [90].

Bioprospecting for new phages is a robust method for discovery of new phages. There are dozens of phages for the four biothreat agents described in this review (Table S1) but other biothreats are lacking in numbers and diversity [91,92]. Expanding the host range of known phages is another strategy that could increase the number of phages available for biothreats. Various approaches have been used to expand the host range of phages [93,94,95,96,97,98,99,100,101,102]. This was demonstrated in T4 phage using an error prone PCR strategy [100] and in T3 phage via site-directed mutagenesis libraries [103] of tail fiber genes, specifically in the host-range-determining regions (HRDRs). Both approaches resulted in mutant phages that were able to infect *Y. ruckeri* and *Y. pseudotuberculosis* strains, respectfully, suggesting these approaches could be used to expand host range to *Y. pestis* as well. By replacing portions of the tail fiber gene gp17 of T3 phage with sequence from *Yersinia* phage R, Ando et al. was able to change the *E. coli* strain host range of these phages to infect and replicate in two *Yersinia pseudotuberculosis* stains [93]. Although not demonstrated, it is likely that this engineered phage host range would also be expanded to *Y. pestis* and could be used for detection purposes. The host range of phages can also be altered by continuous propagation of the phage in the presence of multiple host strains with varying degrees of adsorption when one or more of the strains is restrictive for phage propagation. Using an innovative chemostat system, Holtzman et al. demonstrated that the host range of T7 can evolve to avoid restrictive strains by altering its RBP, even when those restrictive strains have better adsorption properties of the LPS receptor [104]. Host range expansion or changes is also possible for gram positive host specific phages. After identifying and confirming the RBP of the *Listeria monocytogenes* phage PSA, a library of modified PSA phages with mutations in the RBP were generated by error prone PCR, followed by whole genome reconstruction by Gibson assembly [105]. RBP mutants able to shift host serotype specificity were identified and found to have single point mutations in the C-terminal end of the RBP. By creating a chimeric PSA phage encoding both wild-type and mutant RBPs, it was possible to generate phages that had a host range of both serotypes [105]. Furthermore, by mining published sequences of PSA-like prophages, combined with structural knowledge of the RBP, it was possible to redirect phage specificity by exchanging the RBP head domains from these pro-phages with the wild-type domain [105]. These approaches open up the possibility of engineering phages of near neighbor hosts to infect biothreat pathogens that currently have few or no known phages due to their added security and laboratory restrictions constraining phage discovery against these bacteria.

Some phages have the ability to modify their tropism through diversity-generating retroelements (DGRs) encoded by them. This was first demonstrated with the *Bordetella* bacteriophage, BPP-1, which can switch tropism between the infectious and environmental phases of *Bordetella* subspecies [106,107]. By modifying its own RBPs, BPP-1 can produce variants of a small defined region in the RBP that recognize distinct surface receptors of its host [108]. This is accomplished through the use of a reverse transcriptase (RT) encoded in a variability generating cassette. This cassette functions to introduce nucleotide substitutions at 23 sites in a 134-base-pair (bp) variability region (VR) present at the 3′ end of the *mtd* locus, which encodes the RBP. Sites of variability in VR correspond to adenine residues in a nearby homologous template repeat (TR), which is invariant and essential for RT mediated mutagenesis. The process involves transcription of TR to produce TR-RNA, which is then reverse transcribed by the RT to produce TR-cDNA. The TR-cDNA, which has specific errors due to the unfaithful copying of adenines, homes to and replaces VR through an undefined mechanism. This process can theoretically produce 10^13^–10^14^ variable nucleotide sequences at the C-terminal end of the RBP [109,110]. DGRs are found widely among prokaryotes and are not limited to phages [111,112,113,114]. However, DGRs could provide powerful tools for biothreat detection by directed RBP evolution via in vivo DNA diversification [115].

## 3. Conclusions

Biodefense related bacterial pathogens form a unique group due to their notoriety of being highly virulent, easily weaponized, and high consequence nature. Biosurveillance and clinical diagnosis of these pathogens traditionally relied on microbiological, clinical, molecular, and immunological approaches using a variety of platforms and assays. Phage-based detection of these pathogens is still not commonplace and has relied on conventional culture-based plaque assays. Recent more modern rapid approaches include detection of genomic or protein components that exploit the amplification of these components during the active growth of phages inside a given bacterium. Other approaches use reporter phages or very rarely sensor-based approaches. A more comprehensive phage-based rapid and plug and play platform technology for the entire biodefense panel including viruses, spores and toxins is desirable and needed for a unified biodefense posture. Tapping into ongoing revolution in next generation sequencing technologies and other nanosensor platforms will provide a sensitive detection platform for biodefense pathogens. There is an untapped, easy to achieve phage potential that has not been fully exploited despite the fact there is renewed interest in phage therapy around the globe.

## Figures and Tables

**Table 1:** Summary of phage-based methods for the detection of biodefense pathogens.

Organism	Phage/Phage Component/Ligand	Bacterial Target	LOD * CFU/ml	Time to Response	Detection Modality	Reference
*Bacillus* sp.						
	Gamma (γ)	*B. anthracis vegetative cells*	NA	24 h	Plaque assay	[11]
			NA	3–4 h	Omnilog^TM^	[12]
			2.07 × 10^2^	<5 h	PCR	[13]
			1.0 × 10^2^	3–5 h	RT PCR	[14]
			8 × 10^5^	2 h	Lateral Flow Immunoassay	[15]
			1.5 × 10^5^	4 h	Lateral Flow Immunoassay	[15]
	Gamma (γ::*luxAB*), W beta (β::*luxAB*)		1.0 × 10^3^	1 h	Lux reporter Bioluminescence	[16]
	RBP (gp14)::*gfp*		1	~30 min	Fluorescence Microscopy	[17]
	RBPs: γ(gp14), Wip 1 (p23+p24), AP50c (p28+p29), λBa03 (BA4079)::-mCherry		1	~30 min	Fluorescence Microscopy	[18]
	PlyG lysin		1.0 × 10^2^	<5 min	Luciferase assay Bioluminescence	[19]
	PlyG lysin		NA	Hours	Dot blot	[20]
	PlyG Peptide		1–1 × 10^2^	Minutes to hours	Dot blot, ELISA, Q-Dot-Fluorometer and Fluorescence microscopy	[21,22]
	Engineered fd phage with spore binding peptides (landscape phages)	*B. anthracis* Spore	10^3^ spores	30 min	Magnetoelastic micro-resonators	[23]
*Yersinia* sp.						
	φA1122	*Yersinia pestis*, *Y. pseudotuberculosis* **	NA	>24 h	Plaque assay	[24]
	Pokrovskaya					[25]
	L-413C					[26]
	φA1122, L413C	*Yersinia pestis*, *Y. pseudotuberculosis* **	10^3^–10^5^	4 h	Real time PCR	[25,26]
	φA1122::*luxAB*	*Yersinia pestis*	8 × 10^2^	<15 min	Lux reporter Bioluminescence	[27,28]
	RBP φA1122 (gp17)::eGFP		1	<30 min	Fluorescence Microscopy	[29]
	RBP L-413C (gpH)::mCherry		1	<30 min	Fluorescence Microscopy	[29]
	φA1122	*Yersinia pestis*	1 × 10^7^	1 h	MALDI TOF MS system	[30]
*Brucella* sp.						
	Tbilisi (Tb), Firenze (Fz), Weybridge (Wb), S708, Berekeley (Bk), R/C, Izatnagar (Iz)	*B. abortus*, *B.suis*, *B. melitensis*, *B.neotome*, *B. canis*, *B. ovis*	NA	Multiple days	Plaque assay	[10,31,32]
	S708	*B. abortus S19*	10^6^–10^8^	24 h	qPCR	[33]
			10^3^–10^5^	48 h	qPCR	
			1-10^2^	72 h	qPCR	
	Tibilsi (Tb)	*B. abortus*	1 × 10^4^	<1 h	Biosensor-SERS	[32]
*Burkholderia* sp.						
	φX216	*B. pseudomalleii*	3.2 × 10^5^	2 h	MALDI-TOF MS system	[34]

* LOD—Limit of detection; ** Conditional infectivity only for cultures grown at 37 °C; NA: Not applicable; ND: Not determined.

## Supplementary Materials

The following are available online at https://www.mdpi.com/1999-4915/12/12/1393/s1, Table S1: General genomic features of the bacteriophages of biodefense pathogens.
